# Systematic exploration of the ciliary protein landscape by large-scale affinity proteomics

**DOI:** 10.1186/2046-2530-4-S1-P89

**Published:** 2015-07-13

**Authors:** K Boldt, J Van Reeuwijk, Q Lu, K Koutroumpas, N Horn, S Van Beersum, Y Texier, TM Nguyen, JR Willer, E.U. The Syscilia Consortium, N Katsanis, F Képès, RB Russell, M Ueffing, R Roepman

**Affiliations:** 1 Division of Experimental Ophthalmology and Medical Proteome Center, Eberhard-Karls Universität Tübingen, Tübingen, Germany; 2 Department of Human Genetics, Radboud University Medical Center, Nijmegen, The Netherlands; 3 Radboud Institute for Molecular Life Sciences, Radboud University Nijmegen, Nijmegen, The Netherlands; 4 Cell Networks, Bioquant, Cluster of Excellence, University of Heidelberg, Heidelberg, Germany; 5 Institute of Systems and Synthetic Biology, Genopole, CNRS, Université d'Evry, Evry, France; 6 Research Unit Molecular Epigenetics, Helmholtz Zentrum München, Munich, Germany; 7 Center for Human Disease Modeling, Department of Cell Biology, Duke University, Durham, NC, USA; 8 Syscilia, Radboud Institute for Molecular Life Sciences, Radboud University Nijmegen, Nijmegen, The Netherlands

## Objective

Mutations in different ciliopathy-associated genes often result in overlapping clinical phenotypes, which can in part be explained by disruption of overlapping functional protein modules. In this study we conducted large-scale affinity proteomics in a systems biology-based approach to boost insights into the assembly of these ciliary modules, and their connectivity in larger functional protein networks: the ciliary protein interaction landscape. This provides an important framework to deconvolute the pathways and processes that drive ciliopathies, and to understand the general importance of ciliary function for cellular homeostasis.

## Methods

Using more than 220 known and potential ciliary proteins as baits, fused to the Strep/FLAG-tandem affinity purification tag (SF-TAP), we purified protein complexes from human embryonic kidney cells (HEK293T), which were analysed by mass spectrometry. In parallel, specific modules were scrutinized for binary interactions by yeast two-hybrid analyses. Existing and newly developed bioinformatic algorithms were employed to validate the confidence of the identified interactions and to define functional modules.

## Results

We obtained low, medium and high confidence sets of protein interactions and modules. From this data we could assign novel components to known ciliary modules such as the anterograde and retrograde intraflagellar transport modules and the dynein-2 module. Due to the strong focus on ciliary proteins as baits and the integration of data from various sources, we could also identify several new modules, potentially with cilia-associated functions in health and disease.

## Conclusion

Our systems oriented approach, employing affinity proteomics to define the ciliary network has resulted in a comprehensive description of known and candidate ciliary protein networks and modules, which can serve as a resource for candidate ciliopathy proteins and our understanding of pathogenic mechanisms underlying ciliopathies.

